# Sustainable Management of Invasive Algal Waste (*Caulerpa prolifera*): Biomass Compost for Nitrogen Reduction in Vulnerable Coastal Area

**DOI:** 10.3390/plants14243778

**Published:** 2025-12-11

**Authors:** María Carmen Piñero, Carlos García Delgado, Sandra López Rayo, Jacinta Collado-González, Ginés Otálora, Francisco M. del Amor

**Affiliations:** 1 Department of Crop Production and Agri-Technology, Murcia Institute of Agri-Food Research and Development (IMIDA), 30150 Murcia, Spain; jacinta.collado@carm.es (J.C.-G.); gines.oralora@carm.es (G.O.); 2 Department of Geology and Geochemistry, Universidad Autónoma de Madrid, 28049 Madrid, Spain; carlos.garciadelgado@uam.es; 3 Department of Agricultural Chemistry and Food Science, Universidad Autónoma de Madrid, 28049 Madrid, Spain; sandra.lopez@uam.es

**Keywords:** *Caulerpa prolifera*, algal waste, composting, leafy vegetable, nutritional qualities, circular economy framework, antioxidant activity

## Abstract

Composting seaweed biomass reduces environmental impacts while supporting circular-economy strategies in coastal areas, where seaweed removed for recreational management is commonly treated as waste. This approach aligns with regional and EU policies on circular bioeconomy and coastal ecosystem restoration. This study evaluated the effects of Mar Menor seaweed compost applied at 0%, 15% and 35% on lettuce cultivation. Two nitrogen supply levels (100% and 60%) were also used to assess interactive effects on plant growth and nutrient dynamics. The optimal rate of 15% compost enhanced lettuce growth by 25.1% under 100% N irrigation and by 32.2% under 60% N irrigation, indicating that reduced nitrogen availability did not limit biomass accumulation. Indeed, irrigation nitrogen level did not affect total biomass. Compost addition also improved nutrient content and increased phenolic compounds in leaves. When nitrogen was reduced, the combination with compost further boosted phenolic accumulation, by 39.6% with 15% compost and 34.7% with 35%, suggesting a synergistic response. Overall, seaweed compost improves crop performance and nutritional quality while lowering dependence on synthetic fertilisers. Environmentally and economically, it provides coastal municipalities a sustainable option for managing excess seaweed by converting waste into valuable agricultural inputs and mitigating impacts of algal overgrowth.

## 1. Introduction

The Mar Menor, recognised as one of Spain’s largest hypersaline coastal lagoons, has undergone a significant ecological transformation since the 1970s. The modification of the Estación channel facilitated the entry of *Caulerpa prolifera*, a green alga typical of the Mediterranean, which has become the dominant species on the seabed of this lagoon. This seaweed, also known as ‘hare’s ear’, has been classified as invasive due to its ability to alter the natural balance of invaded ecosystems. In the Mar Menor environment, *C. prolifera* forms extensive underwater meadows alongside the marine phanerogam *Cymodocea nodosa* [1,2]. At the end of its life cycle, Caulerpa decomposes and is washed ashore, creating accumulations that give off unpleasant odours. In recent years, the uncontrolled growth of this biomass has led to a considerable increase in algae debris deposited on the shores [3]. In August 2020 alone, nearly 60 tonnes of algae and phanerogams were removed, which entailed a significant expenditure for the regional administration responsible for their management (personal communication).

The treatment of organic waste through composting is an effective and environmentally sustainable strategy that can be applied to various materials, including seaweed [4,5,6]. Compost derived from these seaweeds has significant agronomic properties, as it contains high concentrations of essential nutrients—both macro and microelements—and a high percentage of organic carbon [7]. Its use as an organic amendment reduces dependence on synthetic fertilisers, whose intensive application in agricultural areas such as the Campo de Cartagena has contributed to the environmental degradation of the Mar Menor [8].

In fact, the Mar Menor lagoon is designated as a mass of coastal water affected by nitrate pollution, and was formally established as a “vulnerable zone” for nitrates of agricultural origin in accordance with the European Nitrates Directive (91/676/EEC) [9] and the Law 3/2020, of 27 July, on the recovery and protection of the Mar Menor (BOE No. 221, 17 September 2020) [10]. This recognition implies that specific regulations apply, such as mandatory programs for fertilizer management, prevention of runoff, and control of point and non-point nutrient sources.

In this context, the recovery of algal biomass through composting not only mitigates ecological impacts, but also promotes the implementation of circular economy models in coastal environments, where the systematic removal of seaweeds to preserve the recreational use of the coastline generates significant volumes of waste that are currently managed as refuse. Thus, our study directly aligns with regional and European policy goals for circular bioeconomy and coastal restoration.

The incorporation of seaweed biomass into agricultural systems has shown to have a wide range of beneficial effects on plant development, attributable to its diverse and functional biochemical composition. Among the most notable agronomic effects, we find the activation of germination, increased photosynthetic efficiency, improved water and nutrient absorption, stimulation of flowering and fruiting, delay of senescence processes, and strengthening of defence mechanisms against phytopathogenic agents and abiotic stress conditions such as salinity, drought, or low temperatures. These effects are related to the presence of essential elements such as potassium, nitrogen, micronutrients, humic compounds, functional polysaccharides (laminarin, alginates, and carrageenans) and phytohormones with plant growth-promoting activity [11,12]. However, the agronomic efficacy of seaweeds varies significantly depending on the species used, the time of harvest, and the environmental conditions of the environment of origin, factors that directly affect their chemical and bioactive profile [8,13,14].

Lettuce (*Lactuca sativa* L.), recognised as one of the most important leafy vegetable species globally, stands out for its nutritional profile, characterised by a high concentration of phenolic compounds, water-soluble vitamins, and dietary fibre, attributes that have been associated with preventive effects against cardiovascular diseases [15]. Globally, lettuce production exceeds 27 million tons per year [16]. In the Region of Murcia, this crop represents approximately 28% of the total area devoted to vegetables and up to 65% of national exports [17]. The agronomic and economic magnitude of this production system makes lettuce an ideal model crop for evaluating sustainable fertilization strategies.

In this context, the present study aims to analyse the agronomic and nutritional impact of the application of compost made from algal biomass, by evaluating different percentages of incorporation into the substrate, to optimise the functional quality of the crop and to reduce the use of inorganic nitrogen fertilisers, without compromising the productive yield of lettuce.

## 2. Results and Discussion

### 2.1. Biomass

**Figure 1:**
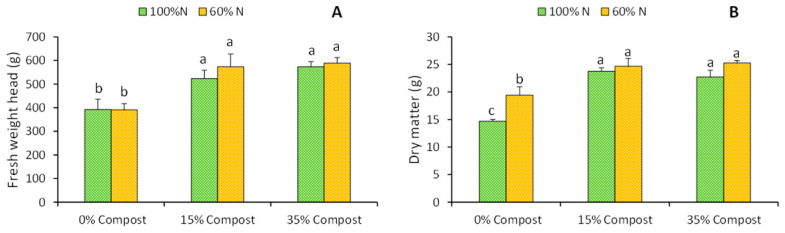
Effects of different irrigation treatments (100% N and 60% N) and different proportions of seaweed-based compost (Control, 15% Compost and 35% Compost) on head fresh weight (**A**) and % dry matter (**B**) of iceberg lettuce. Vertical bars represent mean values ± SE (*n* = 6). Different letters indicate significant (*p* ≤ 0.05) differences between treatments.

In this study, lettuce irrigated with 60% of the recommended N dose did not show a reduction in fresh weight as compared to control plants (100% N) (Figure 1A). Similar results were obtained by Tsouvaltzis et al. [18], who applied an even greater N reduction (50%). In our case, this absence of growth reduction could be explained by the 24.2% increase in dry matter content observed in plants treated with 60% N as compared to the control (Figure 1B). This effect could be attributed to the fact that under conditions of limited nitrogen supply, plants tend to accumulate higher levels of osmotically active solutes, such as nitrates, sulphates, chlorides and soluble carbohydrates, which contribute to maintaining cell turgidity and, consequently, to a higher relative dry matter content [19].

On the other hand, the application of compost formulated with seaweed induced an increase in biomass of around 32.2% in lettuce plants irrigated with 100% N + 35% Compost and in those irrigated with 60% N at both compost proportions, and 25.1% in plants irrigated with 100% N + 15% Compost (Figure 1A). It is important to note that the incorporation of 15% compost—equivalent to an input of 6.22 g of N—was already optimal for promoting significant increases in biomass. In our case, we believe that this improvement in growth was mainly due to the biostimulant effect of the compounds from seaweed, rather than the additional N input, which is consistent with previous literature. Espinosa-Antón et al. [20] reported similar results in tomatoes after the application of seaweed-based amendments. Several studies have attributed these beneficial effects to the bioactive compounds present in seaweed, which can exert a direct action on the primary and secondary metabolism of plants [21,22], also modulating the biosynthesis and accumulation of endogenous metabolites key in the regulation of physiological processes and yield improvement [14,20].

### 2.2. Mineral Content

**Figure 2:**
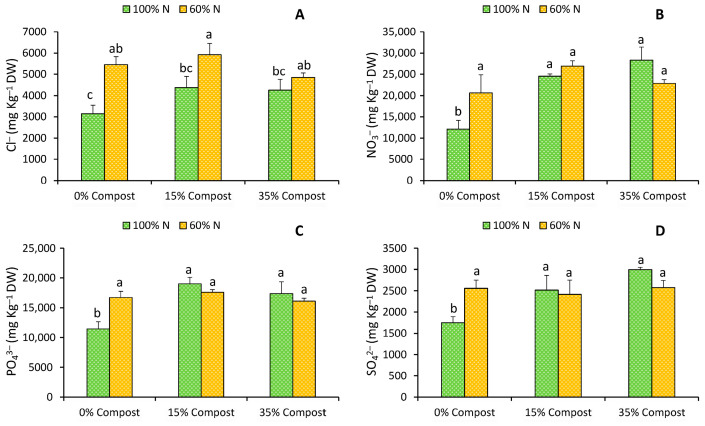
Effects of different irrigation treatments (100% N and 60% N) and different proportions of seaweed-based compost (Control, 15% Compost and 35% Compost) on the contents of chlorides (**A**), nitrates (**B**), phosphates (**C**) and sulphates (**D**) of iceberg lettuce. Vertical bars represent mean values ± SE (*n* = 6). Different letters indicate significant (*p* ≤ 0.05) differences between treatments.

All measured anions (Cl^−^, NO_3_^−^, PO_4_^3−^, and SO_4_^2−^) increased in concentration in lettuce plants irrigated with the 60% N treatment (Figure 2). This increase could be attributed, as previously mentioned, to the tendency of plants subjected to limited nitrogen supply to accumulate osmotically active solutes, which act as osmolytes and contribute to the maintenance of cellular turgor [19].

However, among the analysed cations, only Fe was significantly affected by the 60% N treatment, showing a reduction in its concentration compared with the control plants (Table 1).

In the case of the treatments with compost, when combined with the 60% N treatment, no significant effects were observed on the accumulation of anions in lettuce leaves (Figure 2). However, when combined with 100% N, substantial increases were detected in foliar NO_3_^−^, PO_4_^3−^, and SO_4_^2−^ concentrations, with increases of 50.6%, 39.6%, and 30.3%, respectively, under the 15% compost treatment, and 57.3%, 33.9%, and 41.5% under the 35% compost treatment, as compared with the control (Figure 2). In the case of cations, a similar behaviour was observed. Plants treated with 100% N + 15% compost showed increased Mn (41.4%), Fe (10.0%) and Zn (21.7%) contents as compared to control plants. These results are consistent with those described by Battacharyya et al. [23], Sekhouna et al. [24], and Espinosa-Antón et al. [20], who attributed these increases to the direct action of bioactive compounds present in algae. According to previous studies, these metabolites are associated with physiological processes related to nutrient uptake and utilisation, including the modulation of membrane permeability and the regulation of genes encoding nutrient transporters, together with enhanced biochemical activity at the tissue level and improved leaf development and overall plant performance.

### 2.3. Free Amino Acids

Of the 16 amino acids quantified, those with the highest concentration in lettuce leaves were Tyr, Ser, Ala, Thr, and Arg (Table 2). Regarding the effect of the treatments applied on the amino acid profile, the reduction in N supply caused a decrease in Gly (50.5%), Glu (64.4%), and Tyr (39.0%), while increasing the concentrations of Ser (27.0%), Lys (43.0%), Met (34.1%), and Val (42.8%). Regarding the compost application, the most notable effect was observed in combination with the 60% N treatment, where a significant decrease in Ser, Arg, Asp, Thr, Ala, Pro, Cys, Lys, Met, Ile, and Phe was observed. Considering the behaviour of individual amino acids, it should be noted that Met showed a differential pattern: while its concentration decreased in the 60% N + compost treatment, an increase was observed when compost was applied in combination with 100% N as compared to the control plants. According to Khan et al. [25], Met plays a crucial role in root hair formation and acts as a growth regulator by modulating the action of cytokinins, auxins, and brassinosteroids, thereby promoting root initiation and improving nutrient uptake. Our results suggest, in line with this evidence, the existence of a close relationship between Met content and nutrient concentration in lettuce plants.

### 2.4. Antioxidant Activity, Total Phenolic Compounds and Lipid Peroxidation

**Figure 3:**
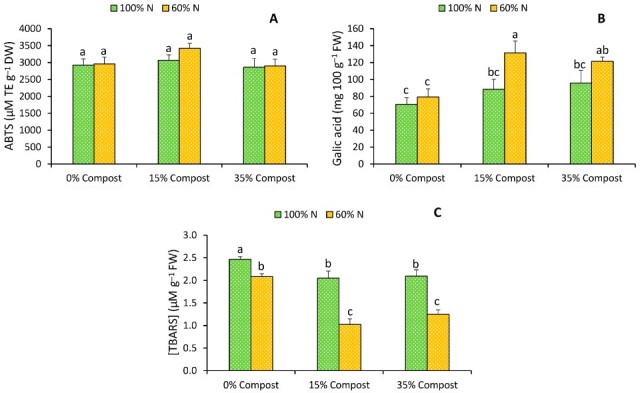
Effects of different irrigation treatments (100% N and 60% N) and different proportions of seaweed-based compost (Control, 15% Compost and 35% Compost) on antioxidant activity (ABTS) (**A**), total phenolic (**B**) and lipid peroxidation ([TBARS]) (**C**) of iceberg lettuce. Vertical bars represent mean values ± SE (*n* = 6). Different letters indicate significant (*p* ≤ 0.05) differences between treatments.

As shown in Figure 3A, antioxidant activity was not significantly affected by either irrigation regime or compost application. However, total phenolic content exhibited a slight increase in plants irrigated with the control solution (100% N) and supplemented with compost, although this rise was not statistically significant (Figure 3B). In contrast, under the 60% N regime, compost application induced a pronounced accumulation of phenolic compounds, with increases of 39.6% under the 15% compost treatment and 34.7% under the 35% compost treatment (Figure 3B). Although the difference in N supply did not affect plant growth, the higher total phenol content observed under the treatment with lower N availability could be due to a metabolic reorientation that increases the relative investment in secondary metabolism pathways. Under conditions of moderately reduced N supply, plants can maintain growth through efficient internal nutrient reallocation; however, the lower demand associated with the synthesis of proteins and other nitrogenous compounds favors the channeling of carbon toward phenylpropanoid pathways, thus promoting greater accumulation of phenolic metabolites [26]. These increases in phenolic content were closely mirrored by the lipid peroxidation responses (Figure 3C). The modest rise in phenolic content observed in plants irrigated with 100% N and supplemented with compost was associated with a slight reduction in lipid peroxidation (16.8% with 15% compost and 14.8% with 35% compost) (Figure 3C). Conversely, the pronounced accumulation of phenolics under the 60% N regime was strongly correlated with a marked decrease in lipid peroxidation, reaching reductions of 50.9% and 40.1% under the 15% and 35% compost treatments, respectively (Figure 3C). These responses could be attributed to the fact that the green algae used in the compost formulation, such as *Caulerpa*, are an important source of secondary metabolites, particularly phenolic compounds, which are subsequently transferred to plant tissue after application [27]. Authors such as Ouahabi et al. [28] have found that *Caulerpa prolifera* contains a wide variety of phenolic compounds, the most abundant being sinapic acid, kaempferol, 7,3,4-trihydroxy flavone, and p-coumaric acid. Phenolic compounds, widely recognised for their role as defence metabolites, play a central role in mitigating oxidative stress by acting as potent free radical scavengers through the donation of electrons or hydrogen atoms, thus interrupting the chain reactions of reactive oxygen species (ROS). Additionally, they contribute to the stabilisation of cell membranes by interacting with membrane lipids, reinforcing their structural integrity and decreasing susceptibility to lipid peroxidation [29].

Various studies have shown that phenolic compounds not only act as antioxidants, but also promote plant development and contribute to improving crop productivity. The scientific literature indicates that these secondary metabolites participate in different stages of the plant life cycle, exerting positive effects on germination, stem and root elongation, biomass accumulation, photosynthetic pigment synthesis, and overall metabolism regulation [30,31]. These results are consistent with our findings, in which compost application was associated with a significant increase in the accumulation of phenolic compounds in leaf tissue, which correlated positively with the increase in fresh weight of lettuce.

### 2.5. Principal Component Analysis

**Figure 4:**
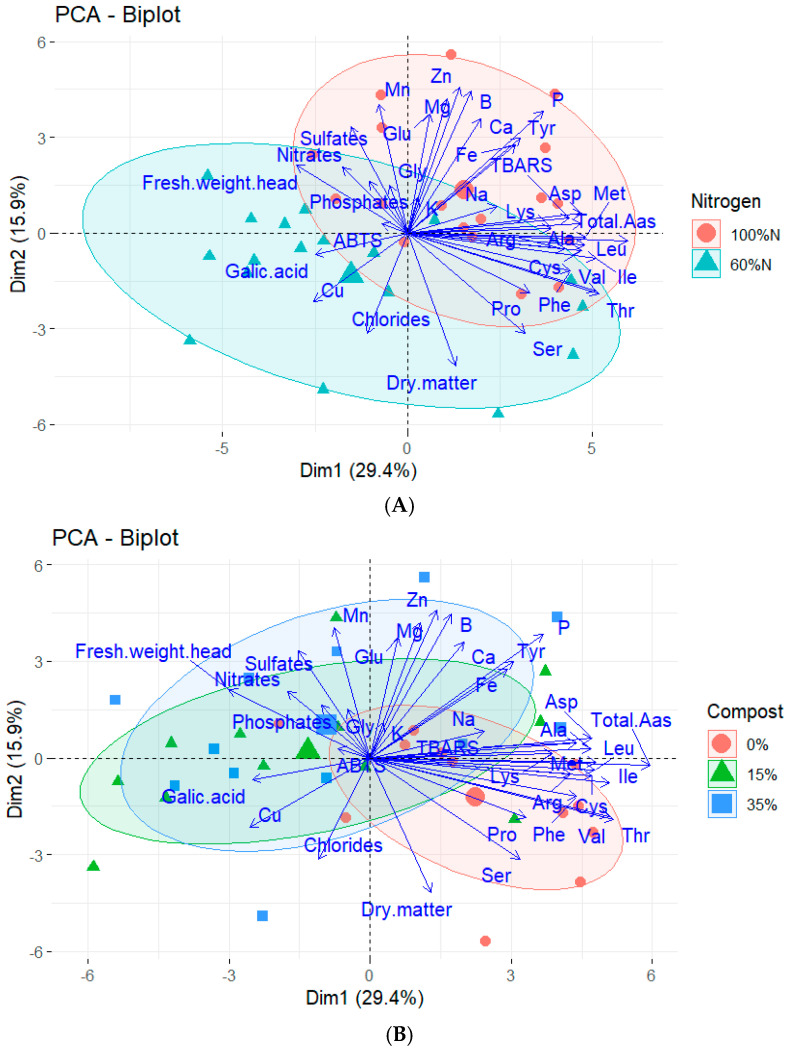
Principal component (PCA) of the parameters analyzed in iceberg lettuce plants grown with different irrigation treatments (100% N and 60% N) (**A**) and different proportions of seaweed-based compost (Control, 15% Compost and 35% Compost) (**B**).

Principal component analysis (PCA) allowed for an integrated visualization of the physiological, nutritional, and metabolic response of the crop to nitrogen fertilization and organic amendments. In the PCA associated with nitrogen levels (Figure 4A), a clear separation was observed between the 100% N and 60% N treatments. Plants grown with 100% N were grouped in the first quartile of the biplot, in close association with most free amino acids (Ala, Asp, Ile, Leu, Val, Phe, Tyr, Met, Lys, Arg, Cys), as well as mineral variables (P, Na, Ca, Fe, B) and the oxidative marker TBARS. This pattern indicates an activation of organic nitrogen metabolism, consistent with greater availability of N for the synthesis of proteins and derived compounds, reflecting a state of high physiological activity.

In contrast, plants with levels below 60% N were grouped in the lower sectors of the biplot, where variables associated with water content and antioxidant metabolism predominated, such as fresh head weight, ABTS, and gallic acid, along with higher concentrations of inorganic anions (sulfates, nitrates, and phosphates) (Figure 4A). This change suggests a metabolic adaptation consistent with increased accumulation of antioxidants and inorganic compounds, possibly as compensatory mechanisms in response to limited nitrogen availability.

In relation to compost treatments (Figure 4B), the separation between groups was more gradual than in the nitrogen PCA, indicating a milder but equally significant modulating effect. The 0% compost was associated with high values of specific amino acids (Ser, Pro, Val, Thr) and higher dry matter content, which could reflect a more restricted and conservation-oriented metabolism. The 15% compost treatment occupied an intermediate position and was associated with increases in antioxidants (ABTS, gallic acid) and mineral anions, suggesting a functional balance between nutritional availability and metabolic responsiveness. On the other hand, 35% compost showed a close relationship with higher micronutrient contents (Mn, Zn, Mg, B, Ca) and increased fresh biomass, pointing to greater mineral availability and stimulation of vegetative growth.

Together, both analyses confirm that variation in nitrogen availability is the most decisive factor in modulating plant metabolism, especially in amino acid accumulation and metabolic activity associated with organic nitrogen. However, the addition of compost acts as an additional modulator capable of readjusting mineral absorption, enhancing antioxidant mechanisms, and promoting growth depending on the level applied. These results highlight the importance of combined mineral and organic fertilization strategies to optimize metabolic functionality and yield in horticultural systems under Mediterranean conditions.

## 3. Materials and Methods

### 3.1. Plant Material and Growth Conditions

Nahifa variety iceberg lettuce seedlings were obtained from a professional nursery (Semillero El Jimenado, S.A., located in El Jimenado, Spain) once they reached a height of between 7 and 9 cm. The young plants were transplanted into 5 L containers containing coconut fibre (Cocopeat, Pelemix, Alhama de Murcia, Murcia, Spain) as the base substrate. A compost derived from seaweed was added to this medium in different proportions (*v*/*v*): 0% (pure coconut fibre), 15% (85% coconut fibre) and 35% (65% coconut fibre). The physicochemical characteristics and elemental composition of the compost are shown in Table 3. Two nitrogen fertilisation regimes were applied: one with full nitrogen supply (100%) as a control, and another with reduced nitrogen supply (60%) to simulate a slight deficiency, while maintaining the levels of the other nutrients identical to the control solution. Irrigation was carried out using a modified Hoagland nutrient solution for the control group, which included the following concentrations: 10.0 mM NO_3_^−^; 1.0 mM H_2_PO_4_^−^; 3.5 mM SO_4_^2−^; 10.0 mM K^+^; 1.8 mM Ca^2+^; 2.2 mM Mg^2+^; 20 µmM Fe; 0.6 µM Cu; 10 µM Mn; 2.0 µM Zn; 40 µM B; 0.5 µM Mo.

As a result, the experimental setup included the following treatment combinations:

(1) 100% N + 0% Compost
(2) 100% N + 15% Compost
(3) 100% N + 35% Compost
(4) 60% N + 0% Compost
(5) 60% N + 15% Compost
(6) 60% N + 35% Compost

To prevent salt buildup and nutrient imbalances in the rhizosphere, an excess volume of nutrient solution was applied throughout the cultivation period, ensuring a minimum leaching fraction of 30% [32]. The study was conducted inside a polycarbonate-covered greenhouse at the IMIDA Research Centre, located in La Alberca (Murcia, Spain). During the experiment, the average daytime and nighttime temperatures were 28 °C and 14.2 °C, respectively, while relative humidity levels averaged 45% during the day and 70% at night.

### 3.2. Method of Composting

The compost was performed by mixing seaweed and urban pruning waste in a ratio 1:2 to assure initial C/N of 30 and adequate structure and porosity. The initial size of the pile was 4 m long, 1.2 m wide, and 0.7 m high. The composting process was performed in windrow pile with periodical turning and irrigation to maintain aerobic conditions and moisture. The composting took place for 163 days.

### 3.3. Biomass

Lettuce plants grown in the compost-amended substrate were harvested 42 days post-transplanting. At this stage, their fresh biomass was measured, and the percentage of dry matter was calculated to assess growth performance.

To determine dry matter, the plant tissue was homogenized and transferred to a duquesita. The fresh weight was recorded, followed by the dry weight after complete removal of water content by freeze-drying.

### 3.4. Mineral Content

To quantify the concentrations of NO_3_^−^, SO_4_^2−^, PO_4_^3−^, and Cl^−^, 0.4 g of lyophilized and finely ground lettuce leaf tissue was mixed with 20 mL of deionized water and agitated for 30 min. The resulting extract was analyzed using ion chromatography (METROHM 861 Advanced Compact IC coupled with METROHM 838 Advanced Sampler) (Metrohm^®^ Ltd.; Herisau, Switzerland), employing a METROHM Metrosep A Supp7 column (250/4.0 mm). Chromatographic conditions included a flow rate of 0.7 mL·min^−1^ and a column temperature maintained at 45 °C. The mobile phase consisted of 3.6 mM sodium carbonate under isocratic elution conditions [15].

For the determination of K, Mg, Na, Ca, Fe, Cu, Mn, Zn, and B, 0.1 g of lyophilized and ground leaf material underwent acid digestion using the ETHOS ONE microwave digestion system (Milestone Inc., Shelton, CT, USA). The digested samples were subsequently analyzed via inductively coupled plasma optical emission spectrometry (ICP-OES) using a Varian Vista MPX instrument (Palo Alto, CA, USA) [15].

### 3.5. Antioxidant Activity, Total Phenolic Compounds and Lipid Peroxidation

The antioxidant capacity was evaluated through the ABTS radical scavenging assay, following the methodology established by Re et al. [33]. For this purpose, 0.5 g of freeze-dried and ground lettuce leaf tissue was extracted with 10 mL of an acidified methanol/water solution (80:20 *v*/*v*, containing 1% HCl) (from Sigma-Aldrich (St. Louis, MO, USA)). The mixture was sonicated at 20 °C for 15 min, stored at 4 °C for 24 h, and then subjected to a second 15-min sonication. Afterward, the samples were centrifuged at 10,000× *g* for 10 min. The ABTS assay was conducted using a calibration curve generated with Trolox (6-hydroxy-2,5,7,8-tetramethylchroman-2-carboxylic acid) (from Sigma-Aldrich (St. Louis, MO, USA)) as the standard. Results are expressed as mean ± standard error in µM Trolox equivalents (TE) per gram of dry weight (DW).

Total phenolic content was determined from lettuce samples previously stored at −80 °C. For extraction, 5 mL of 80% acetone was added to the frozen material. The mixture was then centrifuged at 10,000× *g* for 10 min at 4 °C. An aliquot of 100 µL of the resulting supernatant was combined with 1 mL of Folin–Ciocalteu reagent (diluted 1:10 in Milli-Q water), 2 mL of Milli-Q water, and 5 mL of 20% sodium carbonate solution (both from Sigma-Aldrich (St. Louis, MO, USA)). The reaction mixture was incubated in the dark for 30 min. Absorbance was subsequently measured at 765 nm following the procedure described by Kahkonen et al. [34]. Results were expressed as milligrams of gallic acid equivalents (GAE) per gram of dry weight (mg GAE g^−1^ DW).

Lipid peroxidation levels were assessed by quantifying thiobarbituric acid-reactive substances (TBARS), following the protocol based on the thiobarbituric acid (TBA) assay described by Heath and Packer [35]. For this analysis, 0.1 g of frozen plant tissue was homogenized in 3 mL of 20% (*w*/*v*) trichloroacetic acid (TCA) (from Sigma-Aldrich (St. Louis, MO, USA)). The homogenate was centrifuged at 3500× *g* for 20 min. A 1.5 mL portion of the supernatant was then mixed with 1.5 mL of 20% TCA containing 0.5% TBA and 0.15 mL of 4% butylated hydroxytoluene (BHT) in ethanol (all from Sigma-Aldrich (St. Louis, MO, USA)). The reaction mixture was incubated at 95 °C for 30 min and immediately cooled on ice. After cooling, the samples were centrifuged again at 10,000× *g* for 15 min. Absorbance was recorded at 532 nm, and non-specific absorbance at 600 nm was subtracted. TBARS concentrations were calculated using an extinction coefficient of 155 mM^−1^ cm^−1^ [36].

### 3.6. Free Amino Acids

Free amino acids were extracted from lettuce leaves previously stored at −80 °C. The leaf sap was vortexed at 5000 rpm for 10 min at 4 °C and subsequently filtered through a 0.2 μm nylon membrane. The quantification of free amino acids was performed using the AccQ Tag-Ultra Performance Liquid Chromatography (UPLC) technique [37]. For the derivatization process, 70 μL of borate buffer was added to 10 μL of sap, followed by the addition of 20 μL of reagent solution (from Waters, Milford, MA, USA). The mixture was immediately homogenized and incubated at 55 °C for 10 min. After cooling, a portion of the reaction mixture was injected into the UPLC system. Chromatographic analysis was carried out using an ACQUITY UPLC system (Waters, Milford, MA, USA) equipped with a fluorescence detector (FLR). Separation was achieved on a BEH C18 column (100 mm × 2.1 mm, 1.7 μm particle size [38], operated at a flow rate of 0.7 mL·min^−1^ and maintained at 55 °C. The injection volume was 1 μL, with excitation and emission wavelengths set at 266 nm and 473 nm, respectively. The chromatographic separation was carried out using a binary solvent system composed of two eluents: eluent A, consisting of 5% (*v*/*v*) AccQ·Tag-ultra eluent A concentrate and 95% (*v*/*v*) water; and eluent B, composed of AccQ·Tag ultra eluent B. The gradient profile applied during the run was as follows: from 0 to 0.54 min, 99.9% A and 0.1% B; at 5.74 min, 90.9% A and 9.1% B; at 7.74 min, 78.8% A and 21.2% B; at 8.04 min, 40.4% A and 59.6% B; between 8.05 and 8.64 min, 10% A and 90% B; and finally, from 8.73 to 10 min, the system returned to 99.9% A and 0.1% B. Data acquisition and instrument control were managed using Empower 2 software (Waters, Milford, MA, USA). Quantification of individual amino acids—including serine (Ser), arginine (Arg), glycine (Gly), aspartic acid (Asp), glutamic acid (Glu), threonine (Thr), alanine (Ala), proline (Pro), cysteine (Cys), lysine (Lys), tyrosine (Tyr), methionine (Met), valine (Val), isoleucine (Ile), leucine (Leu), and phenylalanine (Phe)—was performed using certified external standards provided by Thermo Scientific (Waltham, MA, USA).

### 3.7. Statistical Analysis

The experimental design was based on a completely randomised scheme, with six biological replicates per treatment. Statistical processing of the data was performed using Statgraphics Centurion XVI software (StatPoint Technologies, Inc., Warrenton, VA, USA). Before applying inferential tests, the homogeneity of variances and normality of the data were verified using the Kolmogorov–Smirnov test. Subsequently, an analysis of variance (ANOVA) was performed, followed by a comparison of means using Duncan’s multiple range test, establishing a significance level of *p* ≤ 0.05. PCA was applied to reduce data dimensionality and identify patterns among agronomic and physiological variables of lettuce grown with different irrigation treatments (100% N and 60% N) and different proportions of seaweed-based compost (control, 15% compost, and 35% compost). This analysis clearly distinguished the effects of compost made from *Caulerpa prolifera* and nitrogen reduction in the nutrient solution, compared to their respective controls.

## 4. Conclusions

The application of compost produced from Mar Menor algal biomass in *Lactuca sativa* L. cultivation resulted in notable improvements in plant performance. Lettuce grown with the 15% compost treatment exhibited growth increases of 25.1% under full nitrogen supply and 32.2% under reduced nitrogen supply, confirming that nitrogen irrigation level did not influence final biomass production. In addition to enhancing growth, compost application elevated leaf nutrient status and promoted the synthesis of phenolic compounds. When inorganic nitrogen was reduced, this organic amendment further intensified phenolic accumulation—39.6% with 15% compost and 34.7% with 35%—indicating a positive interaction between compost addition and nitrogen input reduction.

These results support the agronomic and environmental potential of using seaweed waste as a valuable resource in agricultural systems, offering an economically viable alternative for coastal municipalities in the management of plant surpluses, while helping to mitigate the impacts of intensive use of synthetic fertilizers and promoting practices aligned with the principles of the circular economy, revaluing a waste product that is currently unused.

## Figures and Tables

**Table 1:** Mean concentrations of cations of lettuce grown with two different irrigation treatments (100% N and 60% N) and different percentages of seaweed-based compost (Control, 15% C and 35% C).

N	Compost	P	K	Ca	Mg	Na		B	Mn	Fe	Zn	Cu
	(C)	g Kg^−1^ DW						mg Kg^−1^ DW				
100%	Control	7.31 ± 0.04 ^ab^	41.74 ± 1.45 ^a^	2.26 ± 0.19 ^a^	1.41 ± 0.02 ^ab^	1.48 ± 0.09 ^a^		16.05 ± 0.37 ^b^	6.40 ± 0.41 ^c^	104.70 ± 2.20 ^b^	55.78 ± 2.03 ^b^	4.07 ± 0.24 ^b^
	15% C	7.62 ± 0.03 ^a^	40.26 ± 0.36 ^a^	1.66 ± 0.23 ^ab^	1.46 ± 0.14 ^a^	1.18 ± 0.09 ^bc^		16.13 ± 0.81 ^b^	10.93 ± 1.51 ^ab^	116.33 ± 4.37 ^a^	71.20 ± 2.41 ^a^	3.22 ± 0.58 ^b^
	35% C	7.59 ± 0.18 ^a^	44.40 ± 1.48 ^a^	2.25 ± 0.20 ^a^	1.40 ± 0.15 ^ab^	1.26 ± 0.08 ^abc^		18.33 ± 1.01 ^a^	13.20 ± 0.53 ^a^	85.38 ± 3.08 ^c^	75.05 ± 5.63 ^a^	3.29 ± 0.58 ^b^
60%	Control	7.14 ± 0.15 ^b^	43.10 ± 1.88 ^a^	1.77 ± 0.25 ^ab^	1.25 ± 0.09 ^ab^	1.41 ± 0.07 ^ab^		15.66 ± 0.34 ^b^	6.07 ± 1.26 ^c^	86.03 ± 2.44 ^c^	54.64 ± 3.03 ^b^	3.97 ± 0.12 ^b^
	15% C	6.77 ± 0.04 ^c^	40.22 ± 1.36 ^a^	1.49 ± 0.43 ^ab^	1.32 ± 0.06 ^ab^	1.10 ± 0.12 ^c^		14.10 ± 0.38 ^b^	6.85 ± 0.14 ^c^	70.70 ± 8.50 ^d^	53.45 ± 0.26 ^b^	5.67 ± 0.08 ^a^
	35% C	6.70 ± 0.11 ^c^	40.85 ± 1.11 ^a^	1.32 ± 0.13 ^b^	1.09 ± 0.18 ^b^	1.08 ± 0.08 ^c^		14.93 ± 1.05 ^b^	9.60 ± 1.83 ^bc^	80.70 ± 2.49 ^cd^	59.93 ± 3.53 ^b^	4.17 ± 0.18 ^b^

The data are presented as the treatment means (*n* = 6) ± SE. Different letters within a column indicate significant (*p* ≤ 0.05) differences between treatments.

**Table 2:** Mean concentrations of amino acids of lettuce grown with two different irrigation treatments (100% N and 60% N) and different percentages of seaweed-based compost (Control, 15% C and 35% C).

[N]	Compost	Ser	Arg	Gly	Asp	Glu	Thr	Ala	Pro	
	(C)	(µmol L^−1^)								
100%	Control	141.0 ± 13.9 ^b^	97.5 ± 28.0 ^ab^	14.3 ± 0.7 ^ab^	47.4 ± 5.2 ^ab^	41.6 ± 4.5 ^a^	110.0 ± 9.2 ^ab^	125.4 ± 15.6 ^ab^	26.1 ± 2.9 ^ab^	
	15% C	129.1 ± 14.7 ^b^	125.2 ± 29.3 ^ab^	9.2 ± 1.4 ^bc^	49.5 ± 3.1 ^a^	37.4 ± 5.0 ^a^	97.4 ± 8.0 ^bc^	180.6 ± 11.2 ^a^	20.3 ± 3.8 ^b^	
	35% C	106.7 ± 13.9 ^b^	104.6 ± 21.0 ^ab^	19.4 ± 3.1 ^a^	31.4 ± 4.5 ^c^	39.1 ± 4.8 ^a^	91.9 ± 16.7 ^bc^	137.8 ± 30.0 ^ab^	22.0 ± 3.4 ^b^	
60%	Control	193.0 ± 15.3 ^a^	183.9 ± 43.2 ^a^	7.1 ± 0.9 ^c^	37.4 ± 1.5 ^bc^	14.8 ± 3.3 ^b^	141.4 ± 17.6 ^a^	161.3 ± 17.3 ^a^	38.4 ± 8.1 ^a^	
	15% C	128.8 ± 19.5 ^b^	78.9 ± 35.1 ^b^	18.0 ± 3.6 ^a^	12.3 ± 2.0 ^d^	23.6 ± 5.2 ^b^	40.9 ± 9.3 ^d^	56.8 ± 4.2 ^c^	24.3 ± 5.0 ^b^	
	35% C	107.8 ± 7.1 ^b^	56.2 ± 8.2 ^b^	10.2 ± 2.2 ^bc^	15.7 ± 2.8 ^d^	20.9 ± 2.3 ^b^	69.7 ± 7.6 ^cd^	87.0 ± 16.0 ^bc^	14.8 ± 2.2 ^b^	
**[N]**	**Compost**	**Cys**	**Lys**	**Tyr**	**Met**	**Val**	**Ile**	**Leu**	**Phe**	**Total**
	**(C)**		**(µmol L^−1^)**							
100%	Control	10.9 ± 0.3 ^a^	14.1 ± 1.4 ^b^	144.1 ± 26.5 ^b^	5.8 ± 0.6 ^b^	61.9 ± 7.8 ^b^	81.0 ± 9.7 ^ab^	71.0 ± 7.0 ^a^	55.0 ± 4.1 ^ab^	1044.6 ± 98.0 ^a^
	15% C	11.4 ± 0.3 ^a^	22.3 ± 3.7 ^b^	149.5 ± 16.4 ^b^	8.8 ± 0.6 ^a^	67.2 ± 11.0 ^b^	92.6 ± 17.9 ^a^	68.9 ± 7.1 ^a^	53.7 ± 3.6 ^ab^	1120.9 ± 102.4 ^a^
	35% C	11.5 ± 0.2 ^a^	30.1 ± 2.5 ^a^	230.5 ± 22.2 ^a^	8.6 ± 1.3 ^a^	63.1 ± 5.0 ^b^	81.2 ± 14.4 ^ab^	73.9 ± 10.1 ^a^	55.1 ± 6.3 ^ab^	1106.6 ± 116.3 ^a^
60%	Control	11.4 ± 0.3 ^a^	24.8 ± 4.2 ^a^	87.8 ± 8.0 ^c^	8.8 ± 0.6 ^a^	108.1 ± 11.9 ^a^	90.4 ± 8.4 ^a^	77.0 ± 5.1 ^a^	68.6 ± 8.7 ^a^	1254.3 ± 91.7 ^a^
	15% C	9.6 ± 0.1 ^b^	5.1 ± 1.0 ^c^	77.2 ± 15.0 ^c^	2.9 ± 0.4 ^c^	24.1 ± 5.3 ^c^	48.7 ± 6.4 ^b^	51.8 ± 10.6 ^a^	49.7 ± 3.9 ^b^	635.4 ± 75.3 ^b^
	35% C	10.1 ± 0.2 ^b^	5.1 ± 1.0 ^c^	105.5 ± 9.5 ^c^	5.4 ± 0.5 ^b^	47.9 ± 6.3 ^b^	55.7 ± 14.1 ^ab^	61.2 ± 6.5 ^a^	48.6 ± 2.3 ^b^	721.7 ± 73.7 ^b^

The data are presented as the treatment means (*n* = 6) ± SE. Different letters within a column indicate significant (*p* ≤ 0.05) differences between treatments.

**Table 3:** Characterization of compost with seaweed remains and coconut fiber.

Parameters	Compost	Coconut Fiber
pH	7.49 ± 0.04	6.3 ± 0.1
EC (µS/cm)	1670 ± 100	225 ± 15
Organic matter	59.20 ± 0.50	80% ± 0.1
%C	28.7	40
%N	2.1	0.5
C/N	13.5	80
Concentrations (mg/kg)		
As	7.42 ± 0.36	Trace levels
Zn	250.67 ± 7.16	9.1
Pb	304.12 ± 5.76	Trace levels
Cd	0.62 ± 0.01	1.06
Cu	9.55 ± 0.84	5.88
P	956.55 ± 49.25	844.00
Mn	970.99 ± 29.24	18.37
Ca	3054.74 ± 30.28	2947.33
Na	903.44 ± 90.68	1387.00
K	1291.57 ± 31.12	6308.00

## Data Availability

The original contributions presented in the study are included in the article, further inquiries can be directed to the corresponding authors.
